# Genome Sequence of *Bacillus subtilis* SPZ1, an Evolved Strain for Higher Uptake Rate of Tributyrin 

**DOI:** 10.1128/genomeA.00511-13

**Published:** 2013-07-25

**Authors:** Ping Song, Xian Xu, Ling Jiang, Renchuang Zhang, Jing Wang, Qing Xu, Shuang Li

**Affiliations:** State Key Laboratory of Materials-Oriented Chemical Engineering, College of Biotechnology and Pharmaceutical Engineering, Nanjing University of Technology, Nanjing, China

## Abstract

The lipase-producing strain *Bacillus subtilis* SPZ1 is isolated from the medium by tributyrin as the sole carbon source. Here, we present a 4.13-Mb assembly of its genome sequence, which may provide various kinds of useful information related to *Bacillus* spp., such as mechanisms and control of the substrate uptake and protein secretion pathways.

## GENOME ANNOUNCEMENT

The genus *Bacillus* is one of the most important groups of industrial microorganisms and is used in several fermentative production processes. *Bacillus* species secrete numerous enzymes to degrade a variety of substrates, enabling them to survive in a continuously changing environment (1). These enzymes are produced commercially, and this production represents about 60% of the industrial enzyme market (2). Thus, research into the mechanisms and control of the substrate uptake and protein secretion pathways will broaden the applications of *Bacillus* as an industrial production host.

In our previous work, the increased tributyrin uptake rate was found to be coupled to a higher lipase production in *Bacillus subtilis* (3). Using tributyrin as the sole carbon source, the adaptive evolution strategy was applied to increase the tributyrin uptake rate. *B. subtilis* SPZ1 was obtained by adaptive evolution with >1,000 generations of growth-based selection from *B. subtilis* CICC20034 (unpublished data). The tributyrin consumption rate of strain SPZ1 reached 0.89 g/(liter·h), which was 1.9-fold higher than that of the original strain. To identify the mutated genes and obtain a deeper profile of the tributyrin assimilation in *B. subtilis*, a series of genetic experiments should be performed.

Here, we present the draft genome sequence of strain SPZ1 using the Illumina HiSeq 2000 next-generation DNA platform, which was performed by Shanghai Majorbio Pharm Technology Co., Ltd., with a paired-end library. The reads were trimmed and *de novo* assembled with SOAP*denovo* (v1.05) (http://soap.genomics.org.cn/soapdenovo.html). Open reading frames (ORFs) were performed by the program Glimmer (http://www.cbcb.umd.edu/software/glimmer/). These ORFs were further annotated by comparison with NCBI-NR and BLASTp (BLAST 2.2.24+). The rRNAs were predicted by RNAmmer (4) and tRNAs were predicted by tRNAscan (5).

The draft genome sequence of strain SPZ1 comprises 4,135,298 bp, which is assembled into 74 contigs. The N_50_ quality measurement of the contigs is 201,909 bp, and the largest contig assembled is approximately 588 kb. It has a G+C content of 46.01%. There are 4,430 predicted protein-coding sequences in the genome sequence. The chromosome has 1 rRNA operon and 52 tRNAs predicted by RNAmmer and tRNAscan, respectively.

The genome sequence of strain SPZ1 serves as a basis for further investigation of the molecular basis for higher tributyrin uptake rate. Further analysis of the genome sequence might also provide other useful information related to SPZ1, such as identifying the genes involved in substrate uptake and protein secretion pathways.

### Nucleotide sequence accession numbers.

This whole-genome shotgun project has been deposited at DDBJ/EMBL/GenBank under the accession no. AQGM00000000. The version described in this paper is the first version, accession no. AQGM01000000.
